# Supplementary material for: High production of furfural by flash pyrolysis of C6 sugars and lignocellulose by Pd-PdO/ZnSO4 catalyst
Source: Nat Commun. 2023 Mar 21;14:1563. doi: 10.1038/s41467-023-37250-0 (PMC10030963; doi:10.1038/s41467-023-37250-0)
Supplement: Supplementary file 1 — Supplementary Information [file 41467_2023_37250_MOESM1_ESM.pdf]

Supplementary Information of

High Production of Furfural by Flash Pyrolysis of C6 Sugars and Lignocelluloses by Pd-PdO/$ZnSO_4$ Catalyst

Qiaoqiao Zhou[1], Jinxing Gu[1], Jingwei Wang[1], Anthony De Girolamo[1], Sasha Yang[1], Lian Zhang[1*]

[1]: Department of Chemical & Biological Engineering, Monash University, Wellington Road, Clayton, Victoria, Australia

[*]: Corresponding author, Tel: +61-3-9905 2592, Email: lian.zhang@monash.edu

A research paper for submission to ***Nature Communications***

Table of Content

1. **Supplementary Figures** 3
2. **Supplementary Tables** 20
3. **Supplementary Note 1** 22
   Mass balance in Pyro-probe reactor 22
4. **Supplementary Note 2** 23
   Mass balance in fixed-bed reactor 23
5. **Supplementary Methods** 23
   Pyroprobe–GC system 23
   Fixed-bed reactor 24
   Cyclic test in Pyro-probe 25
   Sample characterization-acidity measurement 25
   Sample characterization-HTXRD analysis 26
   Sample characterization-TEM sample preparation 26

Supplementary References 27

1. Supplementary Figures

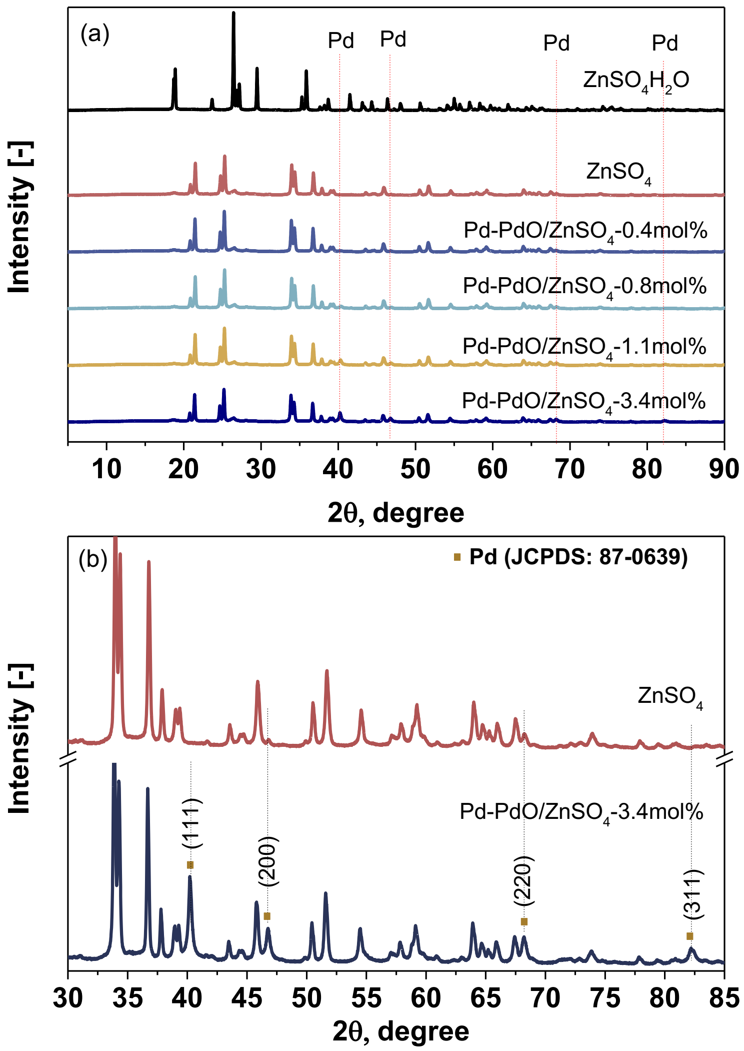

Fig. S1 XRD pattern of different fresh catalysts test in this work. Panel (a) for the reagent-grade $ZnSO_4{\cdot}H_2O$, $ZnSO_4$ and $ZnSO_4$ loaded with different Pd ratios. Panel (b) for an amplified comparison of $ZnSO_4$ standard and 3.4 mol% Pd-doped $ZnSO_4$ catalyst.

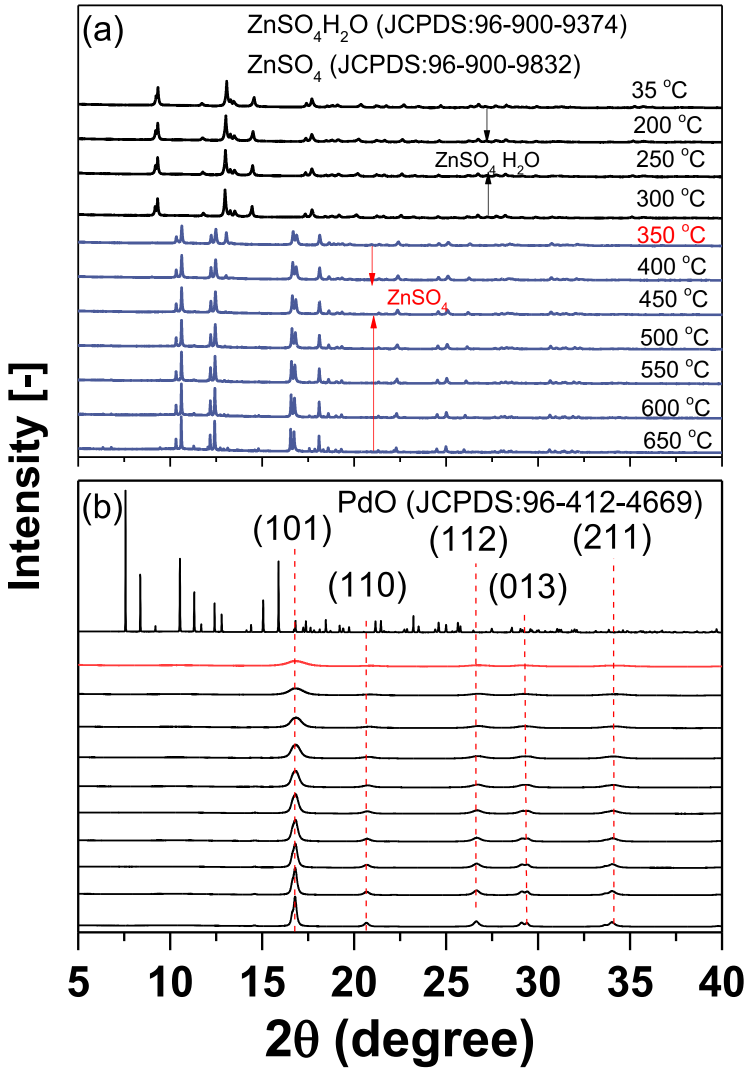

Fig. S2 HTXRD patterns of two pure compounds collected from the temperature-programmed annealing in nitrogen (N_2). Panel (a) for $ZnSO_4 \cdot H_2O$ and Panel (b) for $Pd(NO_3)_2 \cdot 2H_2O$.

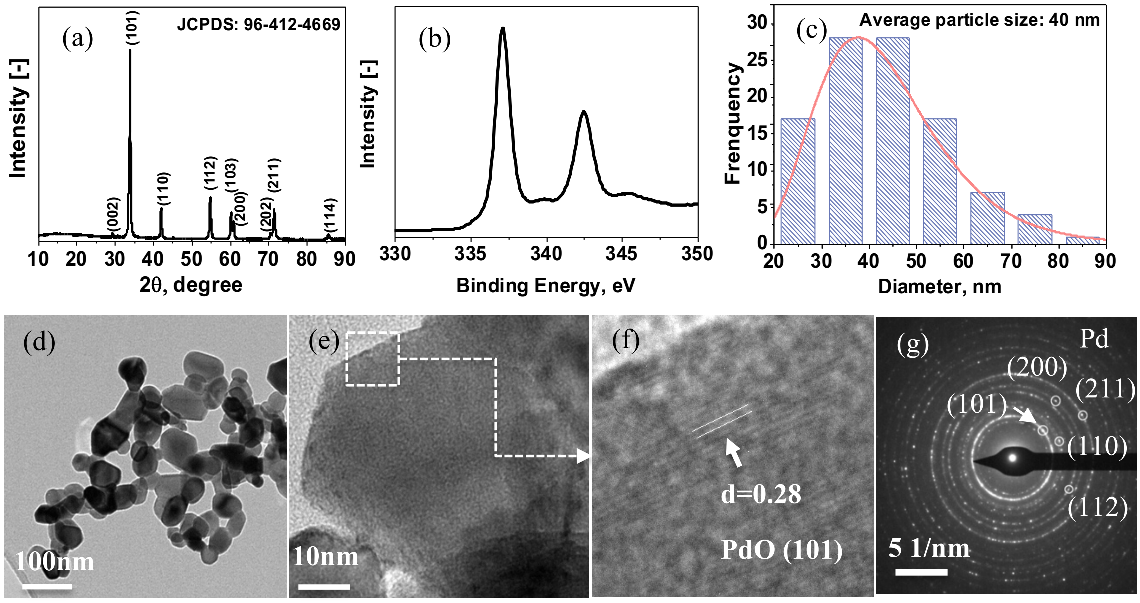

Fig. S3 Properties of the pure PdO crystals. Panels (a) for XRD pattern; (b) for Pd$_{3d}$ XPS spectrum; (c) for particle size distribution; (d)-(f) for typical TEM and HRTEM patterns and (g) for the SAED pattern.

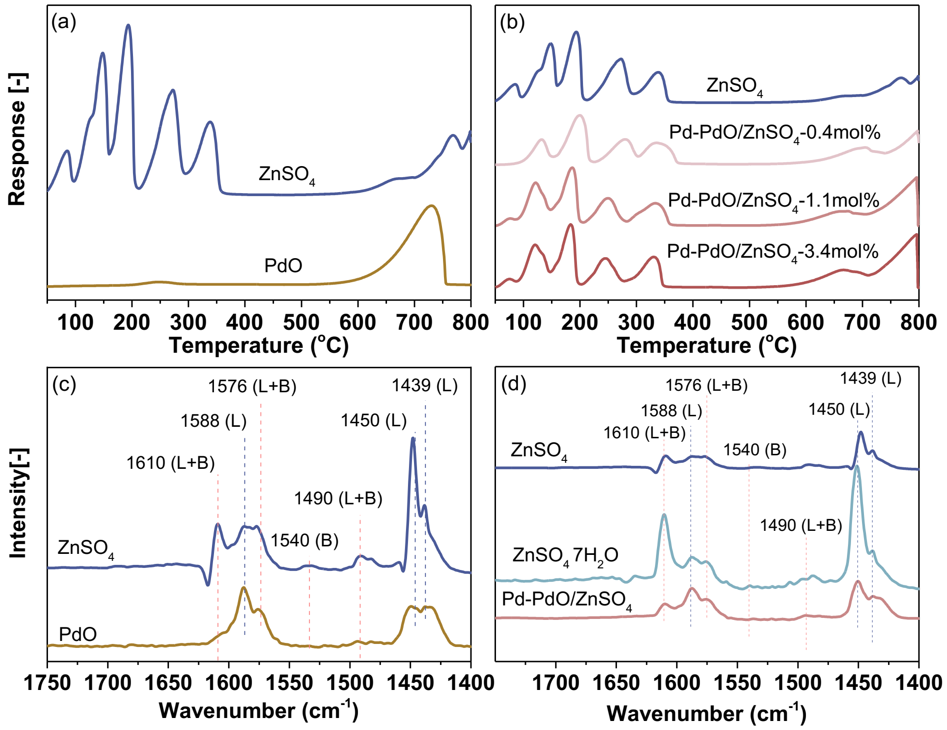

Fig. S4 Acidity of different catalysts. Panels (a) and (b) for NH_3-TPD measured spectra; and (c) and (d) from the Pyridine-FTIR measurement.

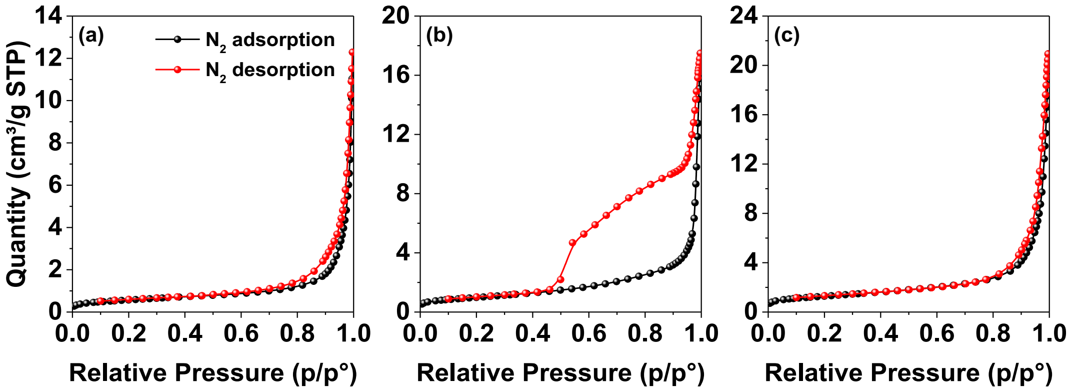

Fig. S5 Isotherm patterns of different catalysts. Panels (a)-(c) for PdO, $ZnSO_4$ and 1.1 mol% Pd-doped $ZnSO_4$ (c) catalyst, respectively.

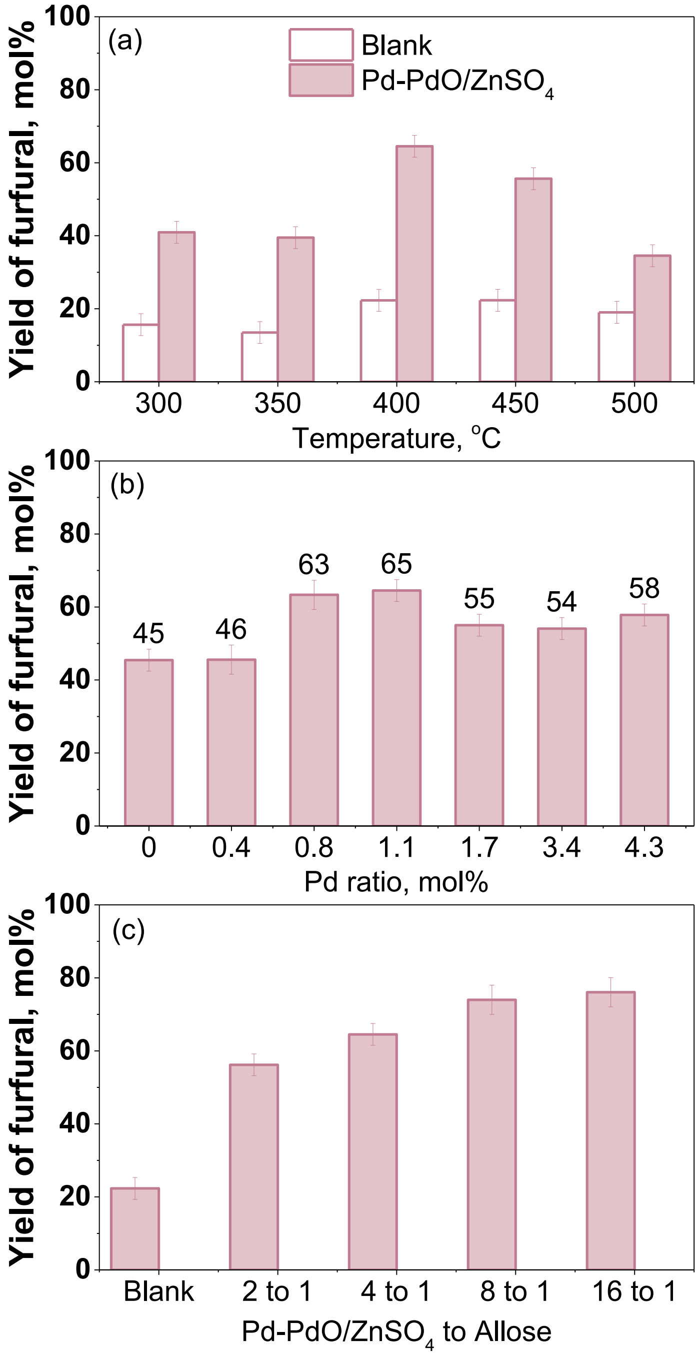

Fig. S6 Production yield of furfural from the pyrolysis of allose with and without the use of catalyst. Panel (a) for the effect of temperature with and without Pd-PdO/$ZnSO_4$ catalyst at a catalyst to allose mass ratio of four. Panel (b) for the effect of different Pd ratios in Pd-PdO/$ZnSO_4$ catalyst at 400 °C at a catalyst to allose mass ratio of four. Panel (c) for the effect of mass ratio of Pd-PdO/$ZnSO_4$ catalyst to allose. Error bars were based on the standard deviation of the results of more than three repetitions.

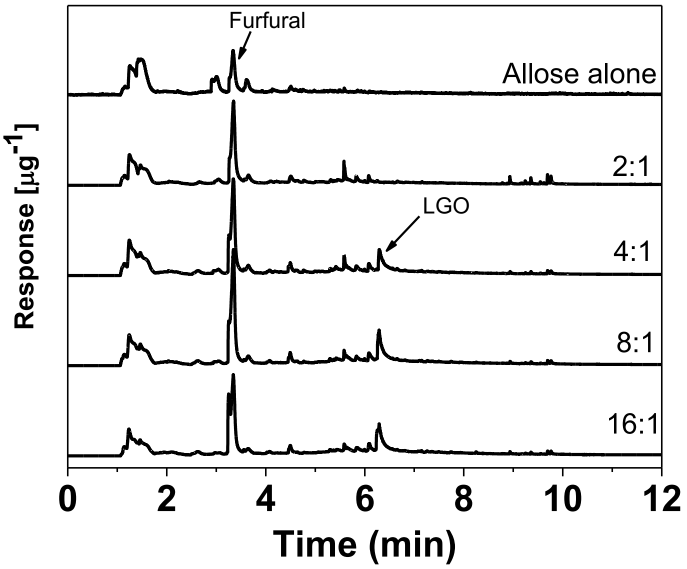

Fig. S7 FID patterns for the liquid products obtained from the pyrolysis of allose with 1.1 mol% Pd-doped $ZnSO_4$ catalyst at different catalyst-to-biomass mass ratios at 400 °C.

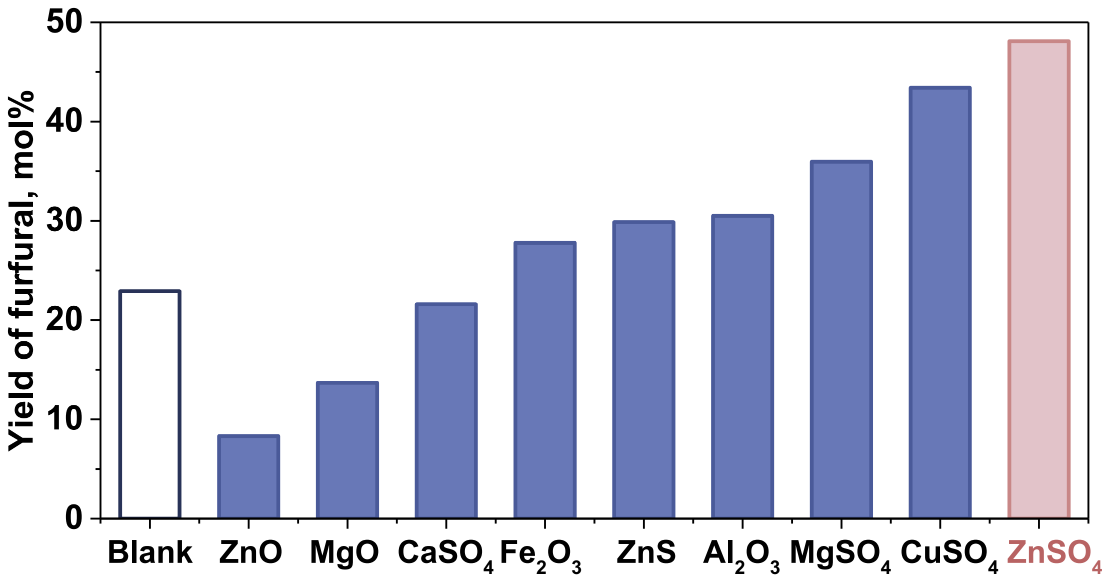

Fig. S8 Production yield of furfural from C6 glucose using different catalysts in Pyro-probe at 400 °C. The catalyst to feedstock mass ratio was fixed at four.

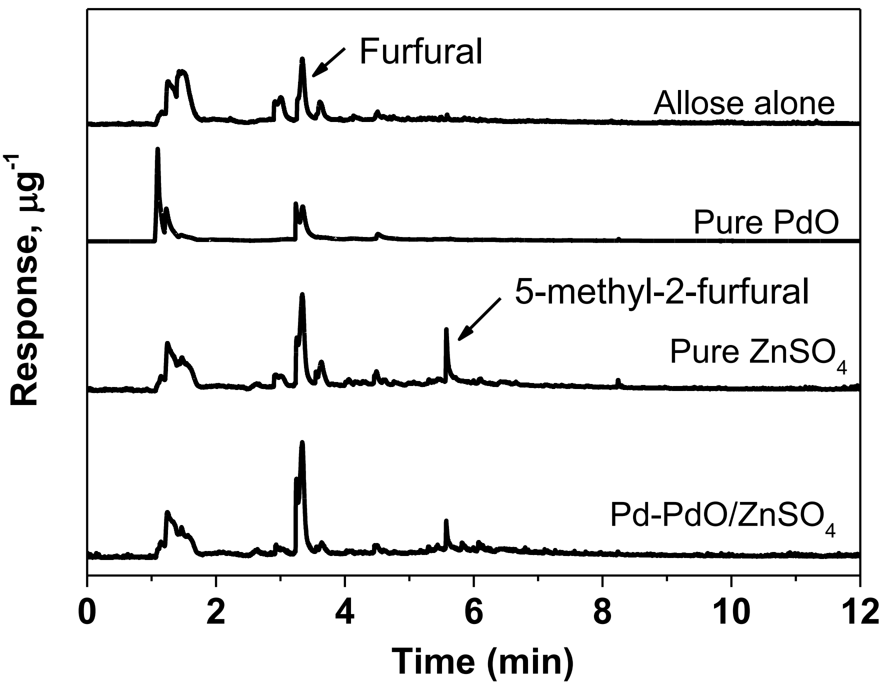

Fig. S9 FID patterns for the liquid products obtained from the pyrolysis of allose with and without different catalysts in Pyro-probe at 400 °C. The catalyst to allose mass ratio was fixed at eight.

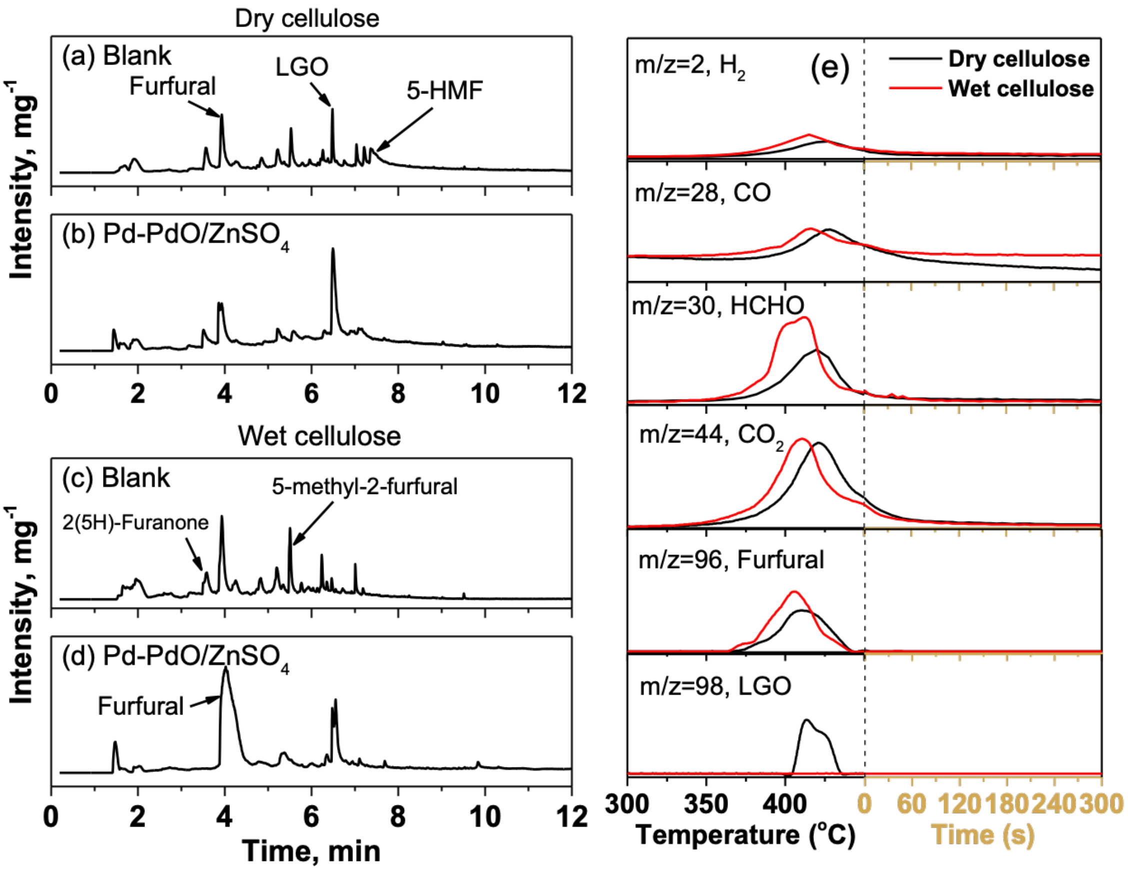

Fig. S10 MS profiles of the liquid products derived from the pyrolysis of dry and wet cellulose. Panels (a) and (b) for the pyrolysis of dry cellulose in Pyro-probe at 400°C. Panels (c) and (d) for pyrolysis of wet cellulose in Pyro-probe at 400°C. Panel (e) for the temperature-programmed pyrolysis of dry and wet cellulose without catalyst in TGA-MS up to 450 °C, and with a hold of 300 secs at 450°C.

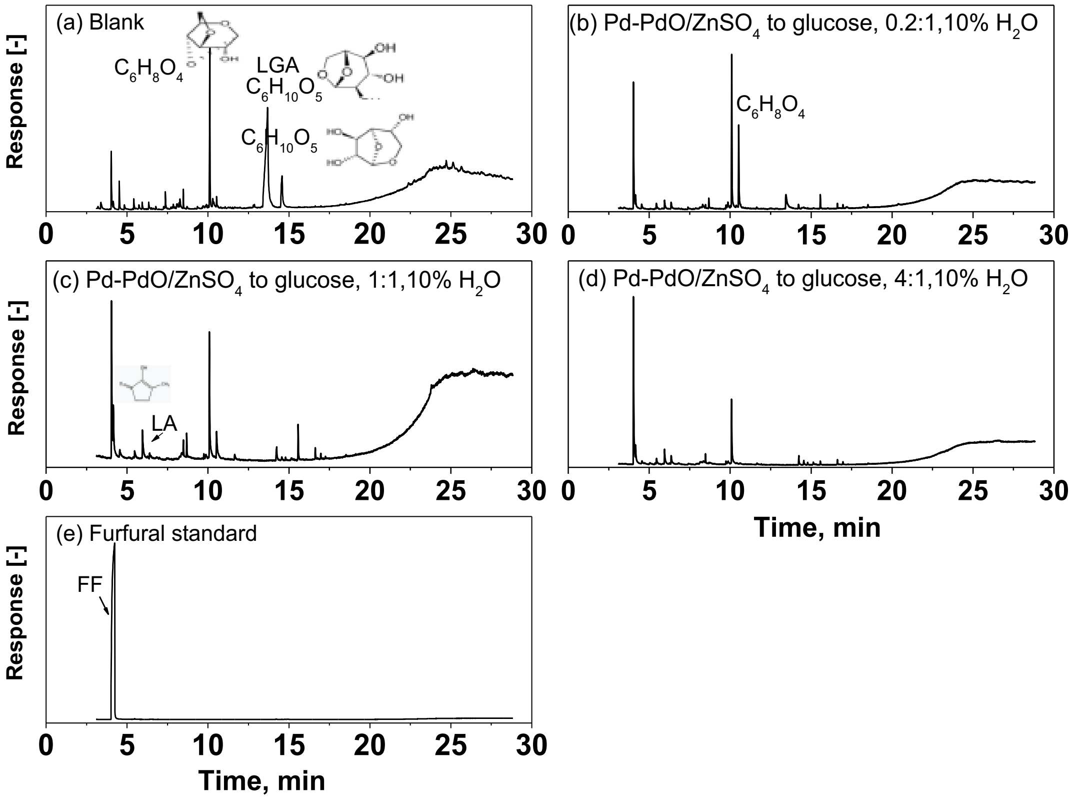

Fig. S11 GC-MS spectra of the liquid product from the pyrolysis of glucose in a lab-scale fixed-bed reactor at 400 °C. Panel (a) for the blank test results in the absence of catalyst and H_2O. Panels (b) – (d) for the results from the use of 10% H_2O and Pd-PdO/$ZnSO_4$ catalyst at a catalyst to glucose mass ratio of 0.2:1, 1:1 and 4:1, respectively. Panel (e) for furfural standard.

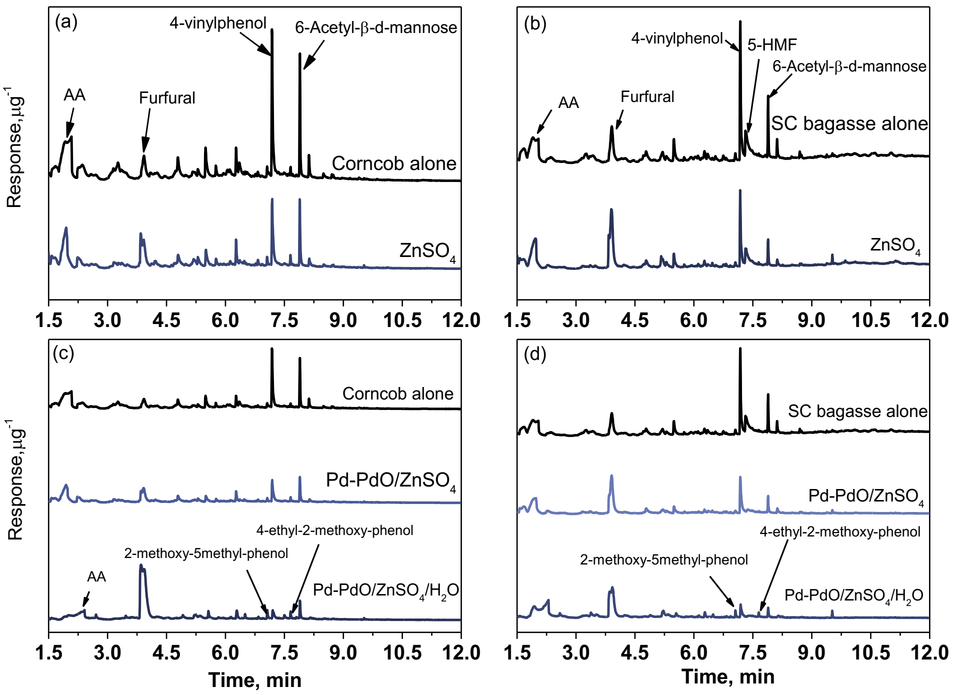

Fig. S12 GC-MS spectra of liquid products from the pyrolysis of real biomass with and without H_2O and different catalysts in Pyro-probe at 400°C. Panels (a) and (c) for corncob. Panels (b) and (d) for sugarcane bagasse. Catalyst to feedstock mass ratio was fixed at eight, and AA stands for acetic acid.

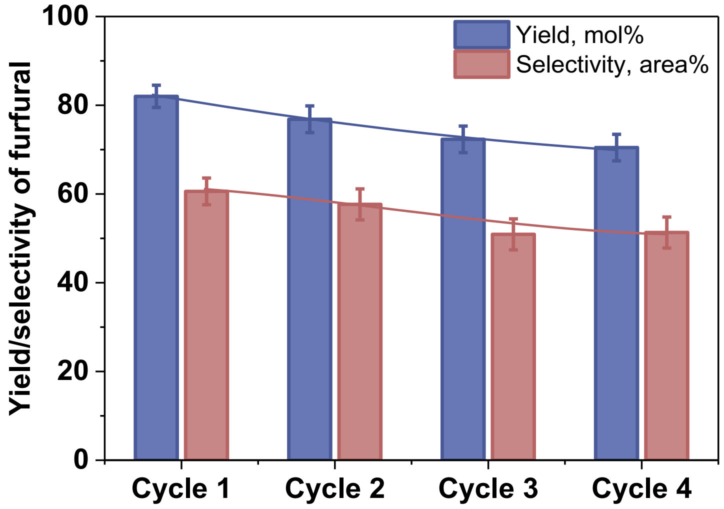

Fig. S13 Selectivity and yield of furfural produced from the pyrolysis of glucose at different cycles at a mass ratio of eight for Pd-PdO/$ZnSO_4$ to glucose 400 °C. Error bars were plotted based on the standard deviation of the results of more than three repetitions.

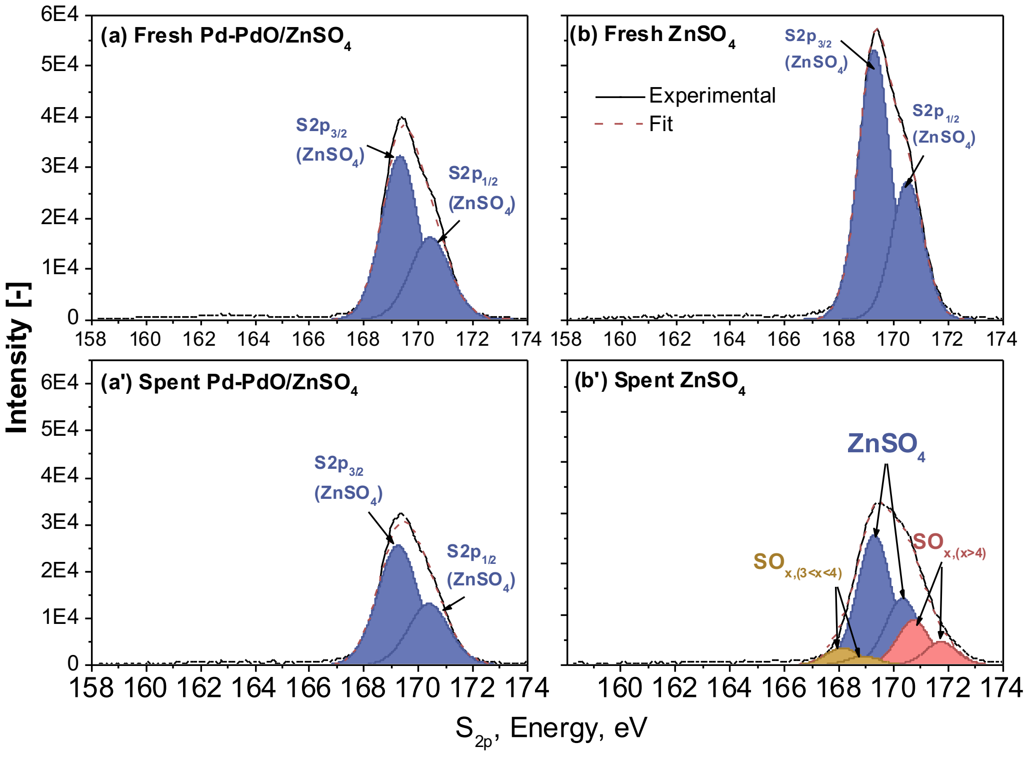

Fig. S14 S*2p* XPS patterns of different catalysts. Panels (a) and (a') for the fresh and spent Pd-PdO/$ZnSO_4$ catalysts, respectively. Panels (b) and (b') for the fresh and spent $ZnSO_4$ catalysts. Spent catalysts were collected from the testing of dry glucose in fixed-bed reactor at 400°C, and with a catalyst to glucose mass ratio of eight.

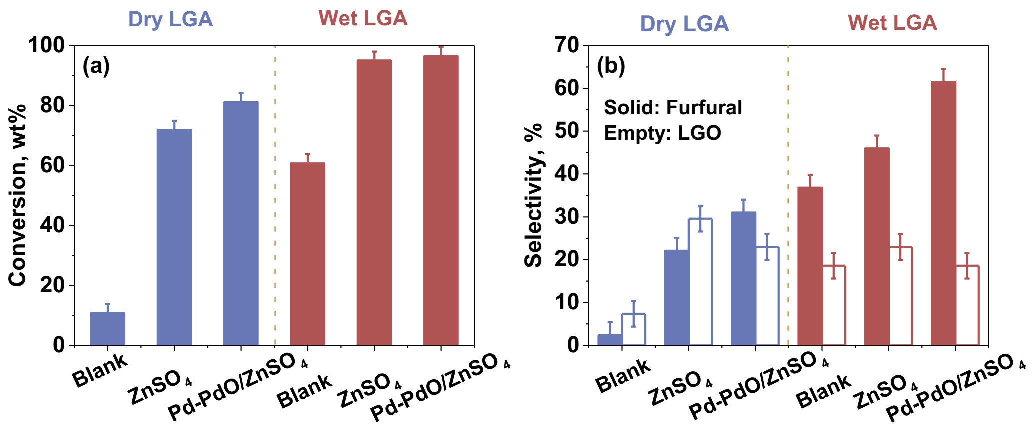

Fig. S15 Conversion of dry and wet LGA with and without the use of catalysts at the catalyst-to-feedstock mass ratio of eight and 400 °C. Panel (a) for the overall conversion of LGA, and Panel (b) for the selectivity of furfural and LGO. Error bars were plotted based on the standard deviation of the results from more than three repetitions.

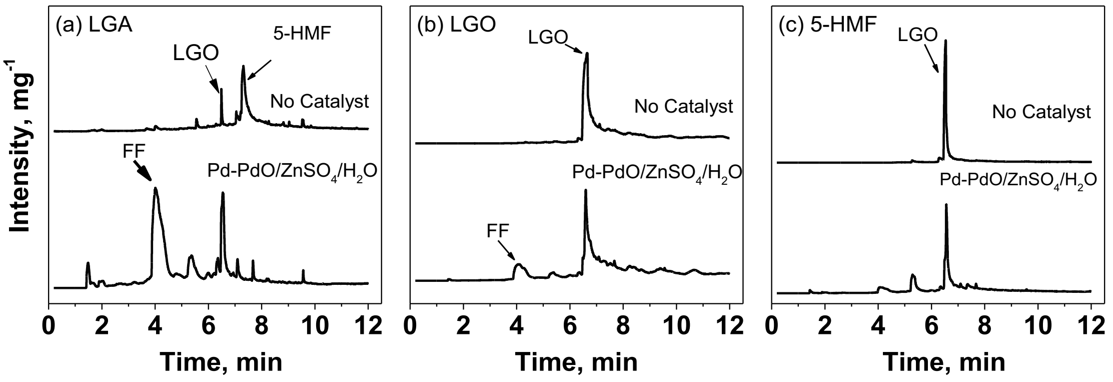

Fig. S16 GC-MS spectra of the liquid products derived from the pyrolysis of three intermediates with and without the use of the Pd-PdO/ZnSO4 catalyst at the catalyst to feedstock mass ratio of eight and 400 °C. Panels (a)-(c) for LGA, LGO and 5-HMF, respectively.

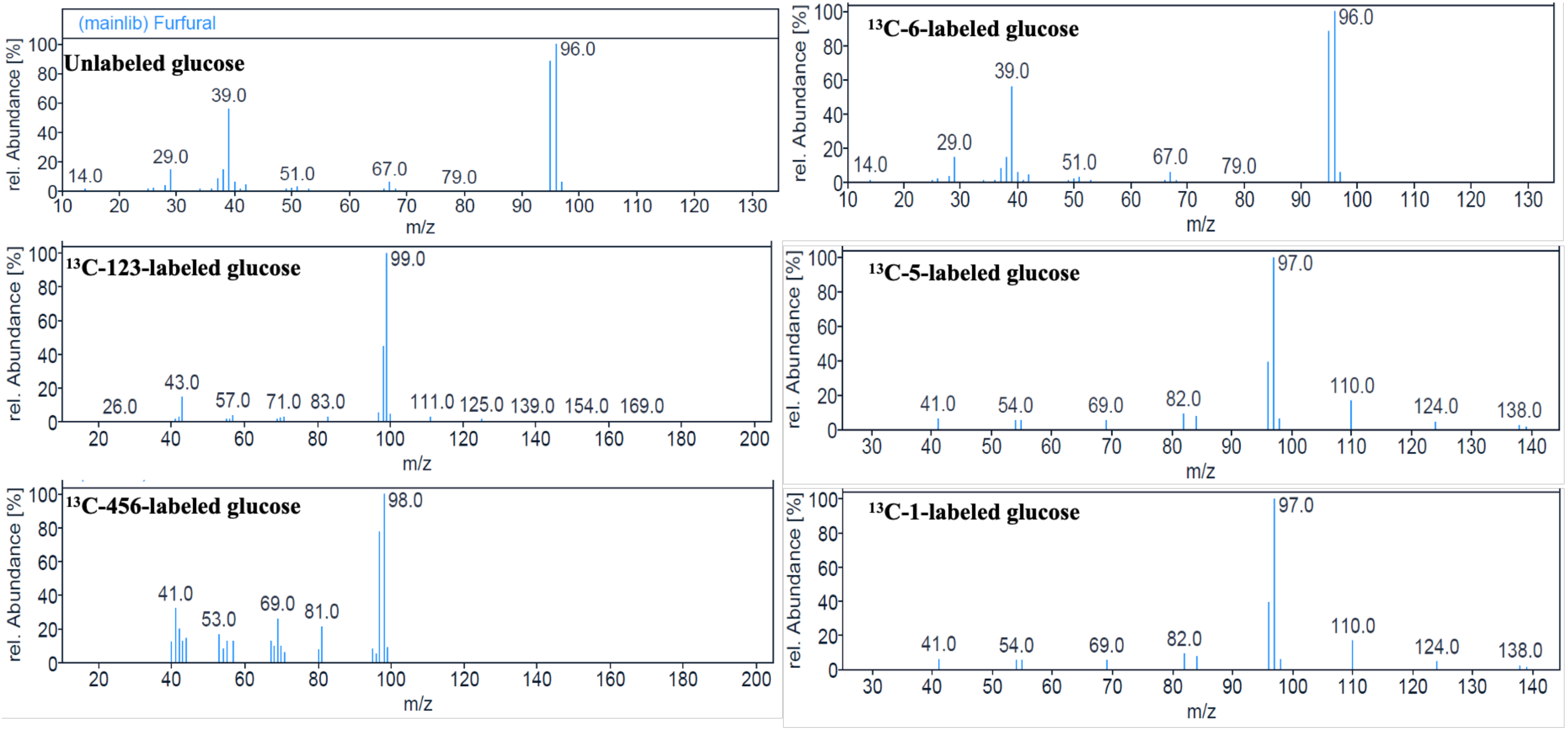

Fig. S17 Mass spectra of furfural produced from unlabeled glucose and different ^{13}C-labeled glucose by Py-GC-MS.

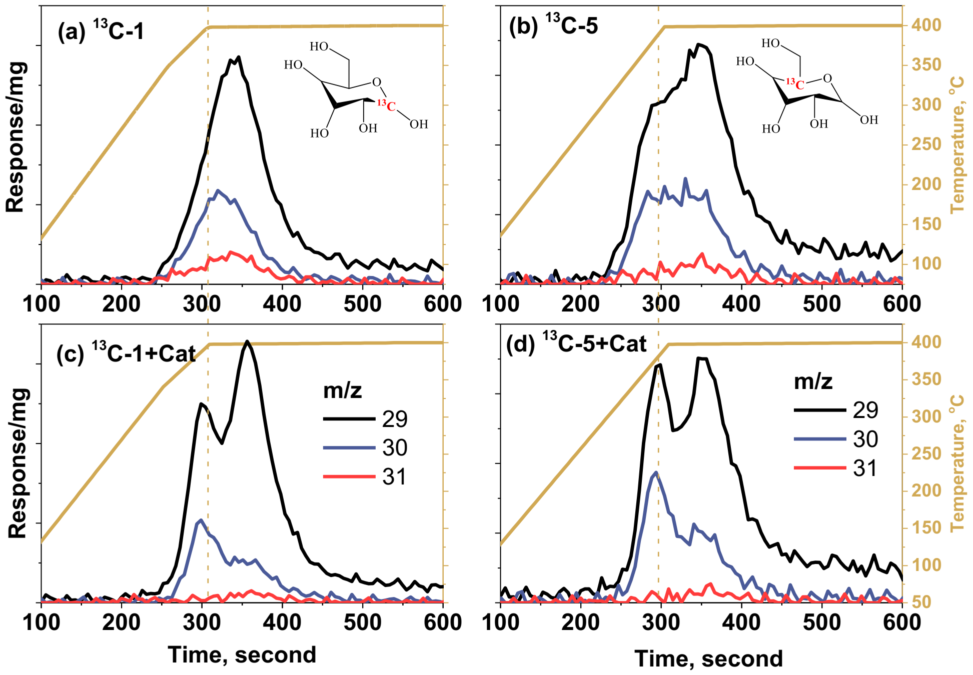

Fig. S18 TGA-MS signals of formaldehyde at m/z of 29, 30 and 31 for ^{13}C-labeled glucose at the C-1 and C-5 positions. Panels (a)-(b) for the absence of the Pd-PdO/$ZnSO_4$ catalyst. Panels (c)-(d) for the presence of the Pd-PdO/$ZnSO_4$ catalyst.

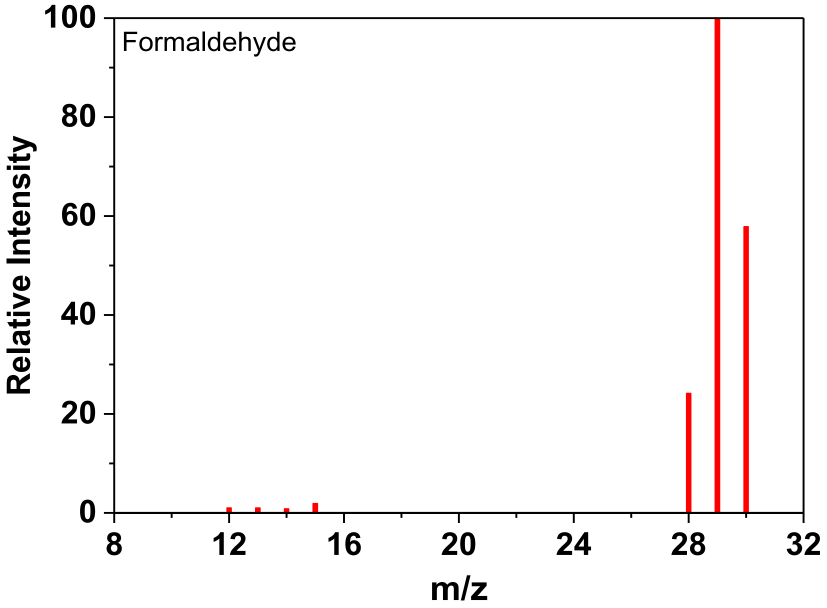

Fig. S19 Mass spectrum of formaldehyde (HCHO) standard.

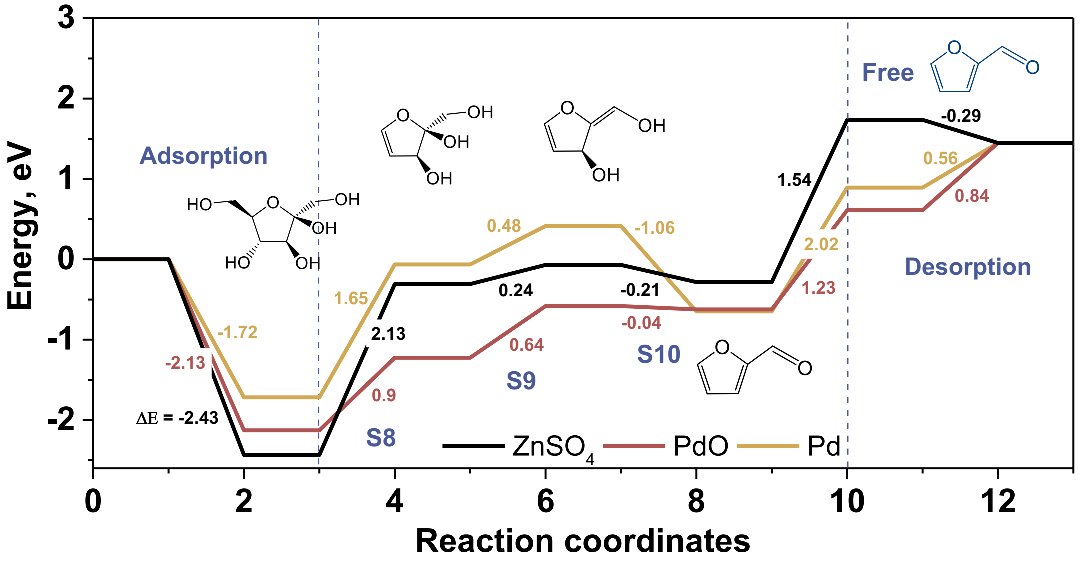

Fig. S20 Energy changes for the formation of furfural on the surfaces of Pd, PdO, and $ZnSO_4$ along the reaction coordinate at -273.15 °C.

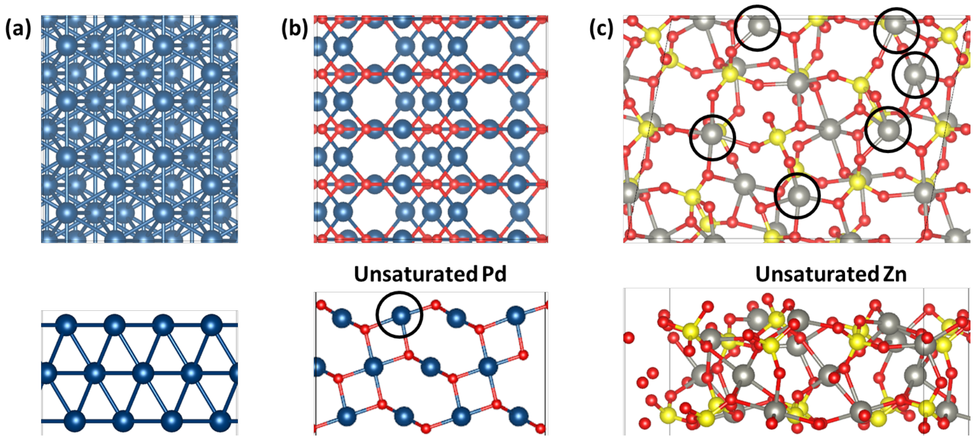

Fig. S21 Optimized slab models used in this work. Panels (a) – (c) for Pd (111), PdO (101), and $ZnSO_4$ (111), respectively.

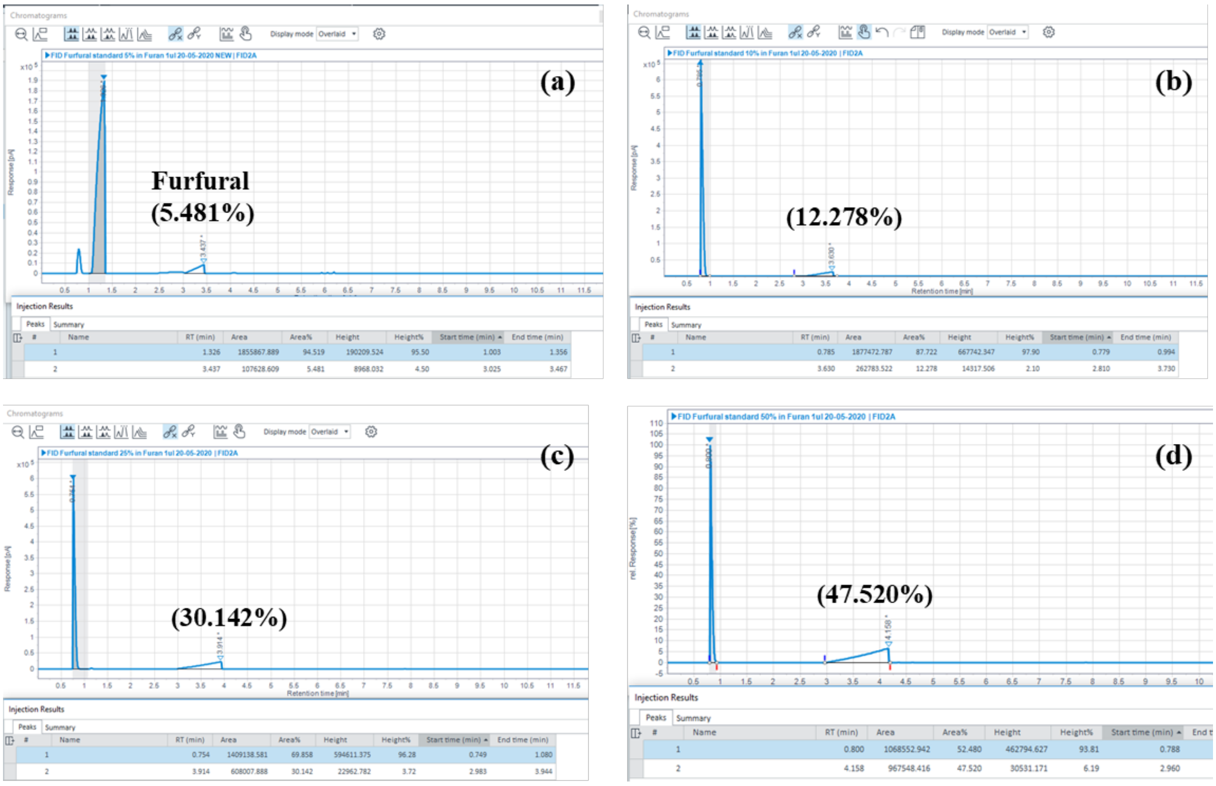

Fig. S22 FID profiles for furfural with different concentrations in furan solvent. Panels (a)-d) for the concentrations of furfural of 6.12 mass%, 12.1 mass%, 29.23 mass% and 55.34 mass%, respectively. Numbers inside the FID profiles refer to the respective area% percentages of furfural.

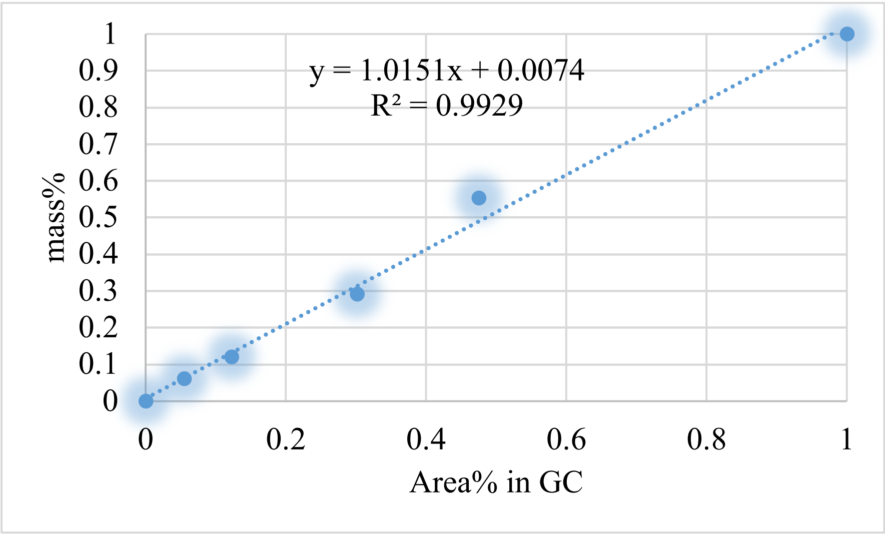

Fig. S23 Correlation of mass% of furfural and its area% from GC-FID analysis in Fig. S22

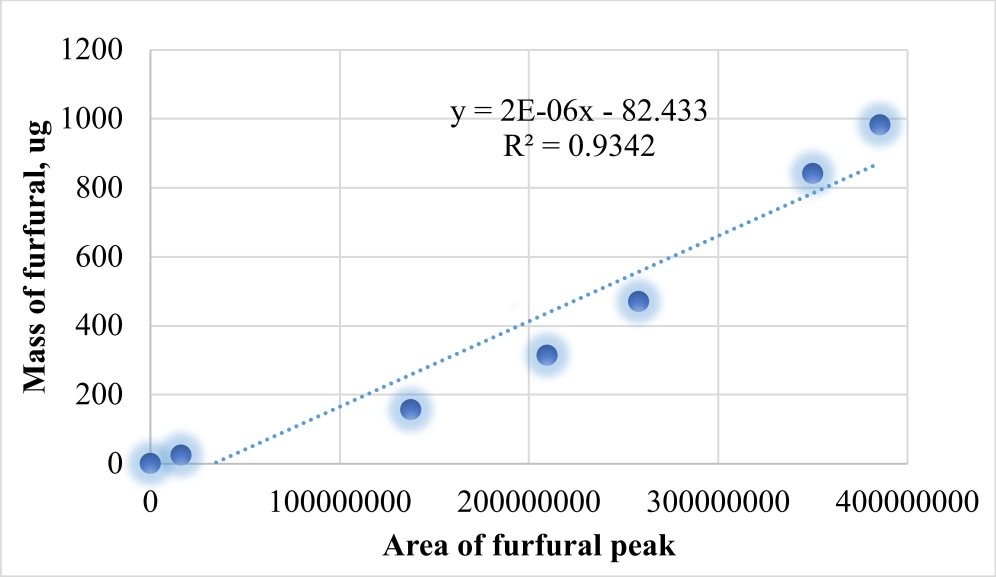

Fig. S24 Calibration curve of furfural based on the absolute mass and area derived from GC-FID analysis in Fig. S22

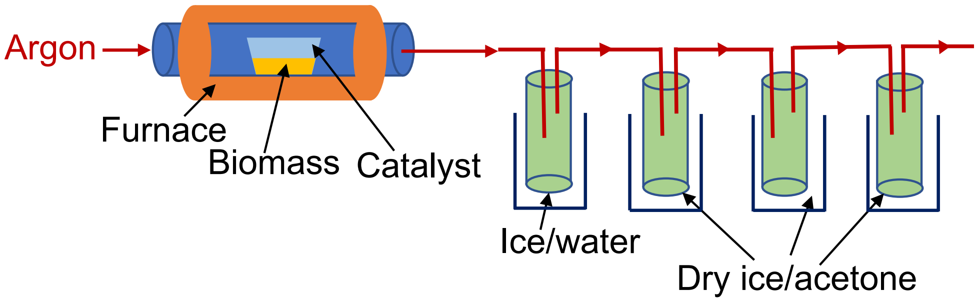

Fig. S25 Schematic diagram of the horizontal fixed-bed pyrolysis reactor

2. Supplementary Tables

Table S1 Selectivity and yield of furfural from the pyrolysis of glucose with and without the use of Pd-PdO/$ZnSO_4$ catalyst and 10% H_2O in a lab-scale fixed-bed reactor at 400 °C

	Catalyst:Glucose mass ratio	Selectivity, %	Yield, mol%
Blank	-	5.2	4.2
Pd-PdO/$ZnSO_4$ with 10% H_2O[b]	(0.2:1)[a]	22.8	30.5
	(1:1)[a]	46.6	62.4
	(4:1)[a]	57.2	76.6

[a] Catalyst to glucose mass ratio;

[b] 10% moisture based on the mass of glucose.

Table S2 Peak assignment summary of GC-FID-MS analysis

Retention time, min	Compound	Molecular formula	Boiling point, °C	Structure
1~2	Light compounds[a]		-	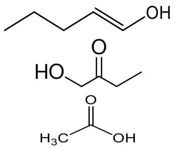
3.3-3.9[b]	Furfural	$C_5H_4O_2$	161.7	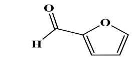
5.2-5.4	5-Methyl-2-furfural	$C_6H_6O_2$	187	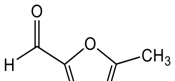
6.4-6.6	LGO	$C_6H_6O_3$	231	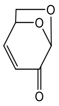
7.1-7.2	DGP	$C_6H_8O_4$	-	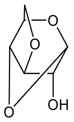
7.3-7.4	5-HMF	$C_6H_6O_3$	114 - 116	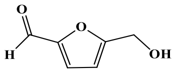
8.1-8.3	LGA	$C_6H_{10}O_5$	385	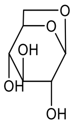
	Glucose	$C_6H_{12}O_6$	-	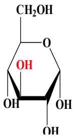
	Allose	$C_6H_{12}O_6$	-	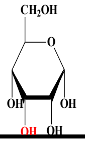

[a] Including pentenol, acetic acid, 1-hydroxy-2-butanone and methyl-furan;
[b] Change in residence time after long-term use of the column.

Table S3 Calibration data for furfural and furan obtained from GC-FID

Furfural			Furan		
Actual mass% in standard	Area in GC	area%	Actual mass% in standard	Area in GC	area%
6.12%	107628.6	5.48	93.88%	1855868	95.50
12.10%	263783.5	12.28	87.90%	1877473	87.72
29.23%	608007.9	30.14	70.77%	1409139	69.86
55.34%	967548.4	47.52	44.66%	1068553	52.48

3. Supplementary Note 1

Mass balance in Pyro-probe reactor

A complete measurement of the mass and yields of all the individual products from Pyro-probe is implausible. However, given the fact that the system is almost leak-free and most of the products are light and easy to flow out, the system is expected to have a reasonably good mass balance. This can be further justified by the case of glucose with the use of the Pd-PdO/$ZnSO_4$ catalyst in Fig 4(d) and (d'). This case has the highest furfural yield, hence, its liquid product is much simpler than the other cases where the presence of other products than furfural in the liquid cannot be calibrated at all.

For the complete conversion of 1 mole glucose ($C_6H_{12}O_6$) into furfural ($C_5H_4O_2$), in principle, it shall follow an overall mass balance of $C_6H_{12}O_6 = C_5H_4O_2 + 3H_2O + HCHO$. The resultant HCHO can further undertake steam reforming to form CO, CO_2 and H_2. For the carbon mass balance, the total amount of CO and CO_2 should be equal to that of furfural, based on the assumption that the secondary reactions of furfural is insignificant. This theory is well supported by the results for glucose in Figs. 4(d) and (d'). As can be seen, on the molar basis

of glucose, 0.773 mole furfural was formed, which is accompanied by ~0.4 mole CO and ~0.38 mole CO_2. The sum of CO and CO_2 clearly agrees very well with furfural. This proves the high accuracy of the Pyro-probe micro-reactor.

4. Supplementary Note 2

Mass balance in fixed-bed reactor

For the mass balance in the fixed-bed reactor, the yields of solid (i.e., biochar), liquid, and gas were calculated separately by their weight, as presented in Eqs. (7)-(9) in the main manuscript. Therefore, the total yield of the products, including gas, liquid and char, can be calculated, which was approximately 100 wt% with a typical error of 5 wt% and a maximum error of 10 wt% [1, 2]. For a typical case of the pyrolysis of 6 g glucose alone, the yields of char, liquid and gas were 21.3%, 58.7% and 17%, respectively. In other words, 97% of the glucose feedstock was measured, which is sufficient for this work.

5. Supplementary Methods

Py–GC system

TCD signals were calibrated by three standard gas mixtures with concentrations of 0.01%, 0.1% and 1% for each gas. The oven temperature was fixed at 50 °C for gas analysis. For the liquid products, the oven was ramped from 50 °C (on hold for 2 min) to 250 °C (on hold for 2 min) at 25 °C/min. The split ratio is set at 25:1. The yield of each gas was calculated by its calibration curve, and thus, the total gas yield, $Y(gas)$, can be obtained.

To quantify the furfural yield, standards with different concentrations of furfural in furan solvent were prepared using a pipette (0 µl-20 µl) and a microgram balance. Their mass percentage and area percentage were calculated and tabulated in Table S3, and the GC-FID

files are presented in Fig. S22. The sample size for GC was 1 μl. The calibration curve for furfural is presented in Fig. S23. The yield of furfural was calculated based on Eq S5. The mass% of furfural or furan is linearly related to the area% of the peak. The standard deviation of the difference between area% and mass% is 3.27% for furfural and 3.26% for furan. Based on these results, we believe that the area% of furfural is representative and proportional to its mass% (error ~3%) for its quantification. Therefore, the selectivity was calculated based on its area% in this work.

Besides, extra efforts have been made to validate the furfural yield using an external calibration method. Around 0-1000 μg furfural was weighed by a micro-balance with an accuracy of 1 μg, and then was injected in the GC-MS. The areas of the furfural peaks were recorded, with their results summarised in Fig. S24. For a typical case using wet corncob feedstock and 1.1 mol% Pd-doped $ZnSO_4$ catalyst, the peak area of the furfural peak in Fig. S12 (c) is 114,545,206, corresponding to a furfural mass yield of 29.6%, which is close to 32.9% shown in Table 2. Due to the complex composition of bio-oil, it is nearly infeasible to calibrate all liquid compounds, therefore, the relative mass percentage calibration method shown in Fig. S23 should be sufficient to compare furfural yields with different catalysts and under different experimental conditions.

Fixed-bed reactor

For each run, the two-stage reactor was preheated to 400 °C in Ar, corresponding to a heating rate of approximately 80 °C/min. After that, approximately 6 g wet glucose (moisture content of 10 wt%) and a certain amount of catalyst loaded on top of glucose in a crucible were inserted into the reactor, as shown in Fig. S25. The mass ratios of catalyst to biomass were 0.2, 1, 2 and 4. The flow rate of Ar was set as 200 mL/min, corresponding to a residence time of 1.5 min.

The discharged effluent was collected via an impinger train, with the ice and water mixture as the cooling agent in the first impinger and the dry ice/acetone mixture in the second to fourth impingers. The permanent gas was sent to a gas analyser (Sensotec Rapidox 5100) for online monitoring of the concentrations of CH_4, CO, CO_2, O_2 and H_2. The liquid bio-oil was then analysed offline by a gas chromatography-mass spectrometer (GC-MS), an HP6890 instrument at a split ratio of 100:1 with 1 mL/min helium as the carrier gas. The oven temperature of GC (capillary column, HP 19091S-433) was held at 50 °C for 3 min and then heated to 300 °C at a rate of 12 °C/min and held for 5 min. The MS (5973 Nexwork) was operated in electron ionization (EI) mode at 70 eV with a scanning mass range of 30-550 m/z. The chromatographic peak area of a compound by GC-MS is considered linear with its quantity [3].

Cyclic test in Pyro-probe

For cyclic testing, the catalyst to glucose mass ratio was fixed at eight, and the temperature was fixed at 400 °C. After each run, the quartz tube reactor was removed from the Pyro-probe and cooled naturally. Afterwards, the spent catalyst was separated from biomass char by a tweezer and saved for the next run. Note that each run was repeated several times to attain a sufficient sample for the next run.

Sample characterization - acidity measurement

The NH_3-TPD analysis was conducted by an AutoChem II 2920. The sample was first treated at 300 °C for 2 h in Ar and then cooled to 50 °C. Subsequently, NH_3 adsorption was performed in a continuous flow of 2000 ppm NH_3/Ar (50 mL/min) for approximately 2 h until saturation. The sample was then heated to 800 °C at 10 °C/min.

Infrared spectra of the catalysts were recorded with a Thermo (Nicolet 380) spectrometer. Sample pellets were prepared under 4-8 MPa with 5 wt% of the catalyst in KBr powder. The background spectrum of the pellet was recorded at room temperature after treatment at 400 °C for 1 h, which was followed by a subsequent evacuation at 10^{-3} Pa for 2 h. The background spectrum was automatically subtracted. To examine the acidity of each catalyst, it was exposed to pyridine at room temperature for approximately 30 min until saturation. Subsequently, the catalyst was evacuated at 40 °C for 1 h, and finally, its spectrum in the wavelength range of 400-4000 cm^{-1} was recorded.

Sample characterization - HTXRD analysis

High-temperature XRD (HTXRD) analysis was performed in the Power Diffraction (PD) Beamline at the *Australian Synchrotron*. The sample was placed in a quartz-glass capillary (1 mm diameter) and protected by nitrogen (N_2) during heating from room temperature to 650 °C at a heating rate of 10 °C/min. The first data were collected at 35 °C. Afterwards, the data were collected from 200 °C to 650 °C at a temperature interval of 50 °C. The temperature was controlled to 1 °C about the setpoint during data collection.

Sample characterization - TEM sample preparation

For sample preparation, a small amount of sample powder was suspended in the butanol solvent. Afterwards, the sample was sonicated to disperse in the solvent and transferred to a holey carbon film supported on a copper grid (300 mesh) using a pipette. Finally, the sample grid was dried in air for approximately 10 minutes prior to analysis.

Supplementary References

1. Zhou Q, Zarei A, De Girolamo A, Yan Y, Zhang L. Catalytic performance of scrap tyre char for the upgrading of eucalyptus pyrolysis derived bio-oil via cracking and deoxygenation. *J Anal Appl Pyrolysis* **139**, 167-176 (2019).

2. Zhou Q, Yang S, Wang H, Liu Z, Zhang L. Selective deoxygenation of biomass volatiles into light oxygenates catalysed by S-doped, nanosized zinc-rich scrap tyre char with in-situ formed multiple acidic sites. *Appl Catal B* **282**, 119603 (2021).

3. Lu Q, Xiong WM, Li WZ, Guo QX, Zhu XF. Catalytic pyrolysis of cellulose with sulfated metal oxides: a promising method for obtaining high yield of light furan compounds. *Bioresour Technol* **100**, 4871-4876 (2009).
